# Early health economic assessment of eLi_12_, a new method to estimate 12-h lithium levels when blood sampling deviates from 12 h

**DOI:** 10.1017/neu.2026.10071

**Published:** 2026-04-29

**Authors:** Ole Köhler-Forsberg, Thea Kirkegaard Kjær, Andrew A. Nierenberg, Tom Bschor, Lars Vedel Kessing, Christian Kraft, Lars Ehlers

**Affiliations:** 1 Psychosis Research Unit, Aarhus University Hospital, Denmark; 2 Department of Clinical Medicine, Aarhus University, Denmark; 3 Nordic Institute of Health Economics A/S, Denmark; 4 Dauten Family Center for Bipolar Treatment Innovation, Massachusetts General Hospital, Harvard Medical School, USA; 5 Departmen of Psychiatry, University Hospital, Technical University of Dresden, Germany; 6 Psychiatric Centre Copenhagen, Denmark; 7 Department for Affective Disorders, Aarhus University Hospital, Denmark

**Keywords:** lithium, bipolar disorder, early health economic assessment, 12-h lithium level, lithium trough level

## Abstract

**Objective:**

Early economic evaluations (EEE) can evaluate the economic potential of new innovative healthcare solutions. We present a methodological framework for EEE in bipolar disorder and use eLi_12_ as an illustrative case, a new method to estimate 12-h lithium blood levels when blood sampling deviates from the 12-h timing, enabling more flexibility for patients and better data on 12-h lithium levels.

**Methods:**

A decision-analytic model evaluated the costs and consequences of eLi_12_ for the treatment of bipolar disorder from a Danish national healthcare payer perspective, assessing the minimum efficacy threshold where eLi_12_ would be considered cost-effective compared with standard of care. The primary outcome was net monetary benefit (NMB), and we estimated quality-adjusted life-years (QALYs) assuming a willingness-to-pay threshold of €67,000/QALY gained. Costs associated with bipolar disorder and lithium treatment (e.g. hospitalisations, suicides, lost productivity, implementation costs) were estimated from literature, Danish registries, and expert opinion.

**Results:**

Assuming 28,000 patients with bipolar disorder whereof 10,000 are treated with lithium, a 2.5% reduction in number of hospitalisations and suicides are sufficient for eLi_12_ to be considered cost-effective within one year of implementation. When using a longer time horizon, allowing more savings to be included and thus considering a smaller improvement to be sufficient, less than 1% improvement by using eLi_12_ would be sufficient within a three-year time horizon.

**Conclusion:**

EEE can evaluate the health economic potential of new innovative methods, supporting early investment decisions and guiding research. eLi_12_ can have significant healthcare savings, emphasising the relevance of studying clinical implementation.

Significant outcomes

- Early health economic evaluations (EEs) can provide an important and helpful framework for the potential health economic savings early during the development process of new innovative solutions.
- eLi_12_ would result in large health economic savings, supporting the clinical potential and emphasising the need to study implementation.

Limitations

- All outcomes are based on expert estimations.
- The actual implementation of eLi_12_ has not been studied.

## Objective

Bipolar disorder is a severe mental disorder posing significant impaired social functioning (Sletved *et al*., 2023), decreased quality of life and an increased risk of suicide (Zhong *et al*., 2024; Yocum & Singh, 2025). Bipolar disorder affects approximately 1–2% (Wittchen *et al*., 2011) and is associated with high healthcare costs, reduced productivity, (Nierenberg *et al*., 2023; Yocum & Singh, 2025), and a need for long-term treatment for several years or even decades (Nierenberg *et al*., 2023; Wiuff *et al*., 2024).

Lithium is the first-line treatment for bipolar disorder and widely used to treat and prevent manic and depressive episodes and maintain long-term stability (Joas *et al*., 2017; Nierenberg *et al*., 2023). Nevertheless, despite the strong evidence base, the use of lithium is low and has been decreasing internationally during the last decades (Kessing, 2024). Although there may be many reasons for this, the requirements of regular control with blood monitoring may complicate the use. Lithium has a narrow therapeutic range, meaning that each patient needs to be on a specific dose (Köhler‐Forsberg *et al*., 2021). The individual lithium dose depends on the specific clinical indication, clinical effect, side effects and lithium blood levels. Guidelines recommend measuring lithium blood levels regularly. In the initial phase, close monitoring is required, while serum level checks at least 2–4 times/year are recommended for long-term treatment to avoid too high or low concentrations, with high concentrations increasing risks for side effects and for lithium intoxication with special risks for the kidneys (Kjaersgaard *et al*., 2012; Kessing *et al*., 2015; Shine *et al*., 2015; Pottegård *et al*., 2016). For >50 years, it has been standard clinical practice that the lithium blood concentration should be measured 12 h after the last lithium dose, with most guidelines recommending a window between 10 and 14 hs (Amdisen, 1967; Marcus *et al*., 1999; Hiemke *et al*., 2011; Licht, 2012; Reddy & Reddy, 2014; Malhi, *et al*., 2016; Yatham *et al*., 2018). However, regular therapeutic drug monitoring is often not performed and many aspects can affect this narrow timing (Nederlof *et al*., 2019; Wiuff *et al*., 2024). Hence, the measured lithium blood level may not reflect the actual 12-h level, which can impact clinical decision-making. Indeed, it has been shown that >75% of lithium-treated patients do not comply with this specific 12-h timing and 50% of lithium blood tests are not even within the 10–14 h window, which likely could affect clinical decision-making and lead to wrong individual lithium dosing (Jacobsen *et al*., 2025).

A stable lithium concentration is an important predictor for good long-term outcomes (Perlis *et al*., 2002; Baldessarini *et al*., 2022), necessitating that P-Li levels are always taken with the same timing. In everyday clinical settings, the main challenge is the patient’s responsibility to time the lithium blood test 12 h after the last lithium dose. If aware of potential wrong timing, the clinician can either estimate a probable 12-h level or the patient needs to take a new blood test to get a correct 12-h level, representing an unnecessary burden on patients, a waste of resources and a delay in treatment decisions. As many lithium-treated patients are followed in non-specialised settings, clinicians may not be aware of potential wrong timing (Jacobsen *et al*., 2025). In response to this clinical challenge, various approaches have been explored to develop methods for lithium-monitoring including point-of-care testing (Sheikh *et al*., 2022). A new method, termed eLi_12_ (estimated lithium level at 12 h), has been developed to estimate the 12-h lithium blood level when the lithium blood test is taken at a different time than 12 h after the last lithium intake, e.g. 8 or 16 h. eLi_12_ presents the clinician with a valid estimate of the 12-h lithium level and holds the potential to improve lithium treatment (Köhler-Forsberg *et al*., 2025). A clinical implementation study is ongoing (NCT07306039).

If eLi_12_ is to be implemented in clinical practice, this requires a thorough assessment of clinical and health economic aspects. Clinical implementation of new solutions, such as eLi_12_, in modern healthcare settings require health EE, representing an internationally standardised method used to inform healthcare decision-makers of the expected value for money of new health technologies (Drummond *et al*., 2015). EEs are widely applied across European countries to support reimbursement and recommendation decisions for new medicines, typically based on best available evidence including data from randomised controlled trials and comparing the costs and consequences relative to the existing clinical practice. EEs are increasingly utilised earlier in the innovation process, before robust clinical evidence on efficacy and safety is available, as they can provide valuable insights into the likelihood of cost-effectiveness. Accordingly, early economic evaluations (EEEs) have been recommended to support investment decisions and guide further research (Hartz & John, 2008; Kim, *et al*., 2020; NICE, 2022). Although EEEs cannot replace full EEs conducted once comprehensive data are available, they represent an important tool for prioritising resources and informing early decisions under uncertainty. Despite previous studies examining current practices in EEEs, a standardised methodological framework for conducting such analyses remains lacking, leading to variability across studies and limiting comparability of results (Hartz & John, 2008; Kim, *et al*., 2020; NICE, 2022).

This study aims to present a methodological framework for an EEE of potential costs and outcomes of innovative technologies in healthcare using eLi_12_ for optimising lithium treatment as an illustrative case. The analysis compares the implementation of eLi_12_ in the Danish healthcare system with standard of care in the management of bipolar disorder, with the objective of identifying the cost-effectiveness threshold for eLi_12_ implementation. Thereby, this study aims to present the usefulness of an EEE approach early during an innovation process of a new data-driven solution for psychiatric clinical care, illustrating the potential benefits of implementation.

## Material and methods

### Early economic evaluation

A decision-analytic model was developed in Microsoft Excel (Microsoft Corporation, Redmond, WA, USA) to evaluate the potential costs and consequences of eLi_12_ for the treatment of bipolar disorder in Denmark. The model was designed from a Danish national healthcare payer perspective to assess the minimum efficacy threshold at which eLi_12_ would be considered cost-effective compared with standard of care. The model calculates the costs and health benefits per average patient and for the entire population of patients with bipolar disorder treated with lithium in Denmark (Table 1). For simplicity, we applied a short time horizon of 1 year as our base-case analysis, selected to adequately capture main costs and effects associated with the intervention in clinical practice assuming 100% implementation. A healthcare sector perspective was chosen to ensure that all relevant direct medical costs and benefits are adequately captured. As sensitivity analyses, we included productivity cost to estimate cost-effectiveness from a broader societal perspective and increased time horizons up to 5 years.

**Table 1.** Baseline characteristics of included patients with bipolar disorders in Denmark treated with lithium

	Mean	Source
Number of patients with bipolar disorder in Denmark	28 000	(Lynge *et al*., 2011)
Number of patients treated with lithium in Denmark	10 000	(Sundhedsdatastyrelsen, 2025)
Age, (years)	51 (SD = 17)	(Lynge *et al*., 2011)
Women, (%)	62	(Lynge *et al*., 2011)
Time since first psychiatric contact, (years)	8.7 (8.58–8.82)	(Köhler-Forsberg *et al*., 2021)
Annual hospitalisation rate (psychiatric care)	19.4%	(Köhler-Forsberg *et al*., 2021)
Number of bed days per year for patients admitted to hospital	59	(Köhler-Forsberg *et al*., 2021)
Reduced life expectancy compared to general population, males, (years)	7.91	(Plana-Ripoll *et al*., 2019)
Reduced life expectancy compared to general population, females, (years)	6.22	(Plana-Ripoll *et al*., 2019)

The primary outcome was net monetary benefit (NMB) (Drummond *et al*., 2015), expressing cost-effectiveness in monetary terms by converting health outcomes into a monetary value. As health outcome, the model estimated quality adjusted life years (QALYs) and assumed a willingness-to-pay (WTP) threshold of €67,000 per QALY gained calculated as approximately 1 × GDP per capita (Woods *et al*., 2016).

The NMB was calculated as: NMB = (∆QALY × WTP)–∆Cost, where *Δ*QALY and *Δ*Cost represent the incremental change in health outcomes and costs per patient from implementing eLi_12_ compared to existing clinical practice. A positive NMB indicates that the intervention is cost-effective at the specified WTP threshold. A negative NMB indicates that no cost-effectiveness.

To identify the efficacy threshold where eLi_12_ implementation would be considered cost-effective, we estimated the NMB for both the total and potential partial improvements in hospitalisation rates, risk of suicide, risk of lithium intoxication, risk of mood episodes, healthcare labour time associated with blood sample handling, and productivity cost. For improvements reaching cost-effectiveness, we estimated the value-based price per blood sample using the same NMB formula. Finally, the model estimated the pay-back period as the number of years before the initial implementation costs and investment for the public payer was fully recovered. To test the robustness of the model, we conducted one-way sensitivity analyses on all inputs in the model. In these analyses, each parameter was varied individually across confidence intervals or plausible ranges while all others were held constant to assess its impact on results (costs, QALYs, and NMB).

### Costs and utilities

The eLi_12_ case model includes relevant, direct costs from a healthcare payer perspective based on the best available literature, official medicines prices, and Diagnose Related Group (DRG) tariffs (Table 2). Danish DRG tariffs are standardised national hospital reimbursement rates that approximate the public hospital’s expected cost for a given activity. Costs associated with lithium treatment and monitoring, hospitalisations, extra lithium monitoring, lost productivity, psychiatrist time spent on interpreting lithium blood samples, and implementation costs were estimated from the literature, Danish health registries, and from expert opinion (OKF, CK, AAN, TB, LVK). This means that we estimated mean improvements of lithium treatment for all patients, independent of whether eLi_12_ would affect the individual treatment course or not, but we included conservative scenarios for these mean improvements.

**Table 2.** Input values applied in the decision-analytic model

	Mean	Source
**Potential improvement in lithium treatment effectiveness from eLi**_12_^*^		
Reduction in probability of hospitalisations	0–15%	Expert opinion
Reduction in probability of fatal suicide	0–15%	Expert opinion
Reduction in probability of poisonings	0–15%	Expert opinion
Reduction in probability of episodes	0–15%	Expert opinion
Reduced time on blood samples (less minutes used per healthcare worker)	0–15%	Expert opinion
Reduced productivity loss from illness (human capital value per year, €)	0–15%	Expert opinion
**Probabilities**		
Hospitalisations (annual risk of admission)	19.4%	(Köhler-Forsberg *et al*., 2021)
Poisonings (annual risk of poisoning)	1.1%	(Ott *et al*., 2016)
Suicides (annual risk of fatal suicide attempt)	0.5%	(Nierenberg *et al*., 2023)
Episodes (annual risk of an episode)	40%	(Severus *et al*., 2014)
**Costs**		
Lithium (€, per patient per day)	1.1	(Lægemiddelstyrelsen, 2025)
Lithium monitoring (€, per patient per year)	470	(PLO, 2025; DRG-takster, 2025)
Hospitalisations (€, per patient admission)	34 261	(Köhler-Forsberg *et al*., 2021; DRG-takster, 2025)
Poisoning (€, per patient)	1952	(Ott *et al*., 2016; Olesen *et al*., 2025)
Implementation costs eLi_12_ (€, total per each of the five Danish hospital regions)	335 000	Expert opinion
Productivity loss associated with illness (€, per patient per year)	33 225	(Vestergaard *et al*., 2020)
Salary psychiatrist (€, per hour)	126.5	(Medicinrådet, 2023; Kommunernes og Regionernes løndatakontor, 2025)
**Utilities**		
Bipolar disorder	0.659	(Hvidberg *et al*., 2023)
Disutility hospitalisations	−0.062	(Zhou *et al*., 2019)
Disutilities poisonings	−0.023	(Porter *et al*., 2024)

* Reduction in the likelihood of annual risk of hospitalisation, suicide, mood episodes.

To have a study cohort representing the basis for all analyses, we used the Danish National Patient Registry to identify all patients diagnosed with bipolar disorder (ICD-10: F30-31) at a Danish psychiatric in- or outpatient setting in the period from 2005 to 2023 and who were alive on December 31, 2023 (Lynge *et al*., 2011). The annual hospitalisation rates were identified for all individuals with bipolar disorder during the same period. The sex distribution and mean age were identified on December 31, 2023. To identify the number of lithium (ATC code N05AN01) users in Denmark in 2024, we used the openly available homepage www.medstat.dk, which has registered accumulated data on drug use in Denmark (Sundhedsdatastyrelsen, 2025). All costs were estimated in Danish Krone adjusted to price year 2025 and subsequently converted to Euro (€) using the exchange rate of 7.46. Utilities and disutilities were measured by EuroQoL-5 Dimension 3 Levels (EQ-5D-3L) data from published sources.

## Results

The number of patients with bipolar disorders in Denmark during 2005–2023 was 28,000, of whom approximately 36% are treated with lithium (i.e. 10,000 lithium users in 2024 according to www.medstat.dk) (Table 1). Mean age was 51 years (SD = 17) and 62% were females. Based on the input variables for the decision-analytic model (Table 2), we present the annual reduced probabilities for all outcomes including the NMB within a one-year timeframe for all outcomes based on the different scenarios of improvement of lithium (Table 3, ranging from 1% to 15%). 0% effect of eLi_12_ on all investigated outcomes after one year equals a NMB at –1,675,000 €.

**Table 3.** Potential effect of eLi_12_

Potential effect of eLi_12_							
		1%	2%	3%	5%	10%	15%
Number per year	Reduced probability of hospitalisation (Bed days avoided)	1079	2159	3238	5396	10792	16189
	Reduced probability of fatal suicides (Suicides avoided)	0	1	1	2	4	6
	Reduced probability of poisonings (poisonings avoided)	1	2	3	5	10	15
	Reduced probability of episodes (Episodes avoided)	38	76	113	189	378	566
	Reduced time on clinical evaluation (total minutes per evaluation)	80 000	160 000	240 000	400 000	800 000	1 200 000
	Reduced proportion with productivity loss (number of people)	94	189	283	472	944	1416
NMB (€), 1-year horizon^*^	Reduced probability of hospitalisation	−975 000	−275 000	426 000	1 826 000	5 327 000	8 828 000
	Reduced probability of fatal suicides	−1 659 000	−1 644 000	−1 628 000	−1 597 000	−1 519 000	−1 442 000
	Reduced probability of poisonings	−1 671 000	−1 668 000	−1 664 000	−1 657 000	−1 639 000	−1 621 000
	Reduced time on clinical evaluation	−1 506 284	−1 337 568	−1 168 852	831 420	12 159	855 739
	Reduced proportion with productivity loss	1 461 000	4 598 000	7 734 000	14 007 000	29 689 000	45 371 000

* NMB on reduced probability of episodes are not included separately as it included in calculations for hospitalisation and productivity.

**Figure 1.**
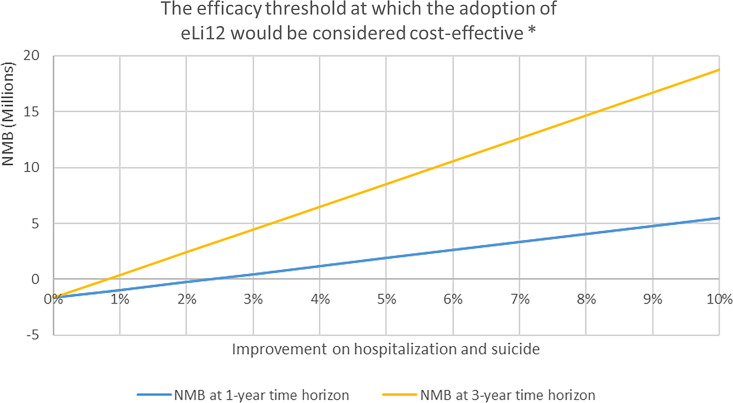
The efficacy threshold of eLi_12_*.

The main results showed that a 2.5% reduction in number of hospitalisation and suicides will be sufficient for eLi_12_ to be considered cost-effective within one year from a national payer perspective (Figure 1). Including a longer time horizon would allow more savings to be included, and thus a smaller improvement would be sufficient, with less than 1% effect being sufficient with a three-year time horizon. Partial improvements in suicide rates were insufficient alone to reach cost-effectiveness within the 5-year horizon (Table 3).

**Figure 2.**
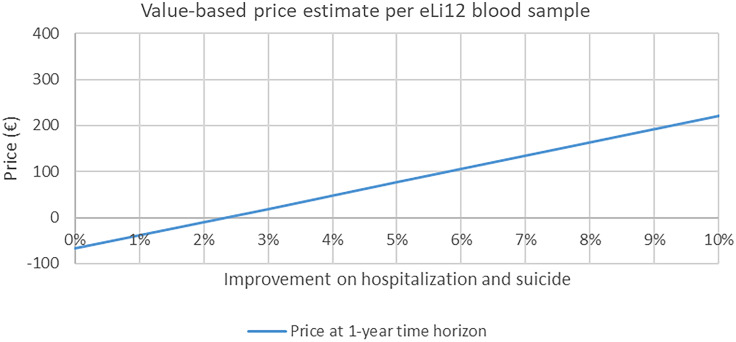
Value-based price of eLi_12_ at different levels of effectiveness*.

**Figure 3.**
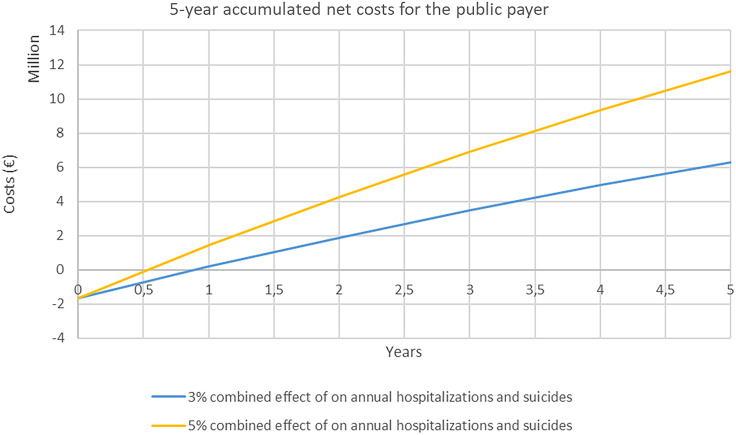
Payback time for the public payer*.

Assuming a 3% reduction in hospitalisation and suicides, a ‘value-based price’ for eLi_12_ would be around €19 per blood sample (Figure 2). At this level of effectiveness of a 3% reduction in hospitalisations and suicides, the public health sector would be able to cover the initial costs of implementation in 1 year after which, there will be accumulated net savings for the payer (i.e. in Denmark, the public Regions and university hospitals) (Figure 3). Increasing the effectiveness of eLi_12_ to 5% reductions in hospitalisations and suicides reduced pack-back time to approximately 0.5 years.

Sensitivity analyses indicated that results were uncertain due to lack of precise evidence of effectiveness of eLi_12_, especially inputs affecting the potential gain in hospitalisation and QALY (data not shown).

## Discussion

This study presents a methodological framework for an EEE of a new innovative solution that can be used in patients with bipolar disorder treated with lithium. This EEE approach can be useful and help decision-makers to evaluate the potential economic advantages of a new solution. The eLi_12_ solution, which was used as a case, was shown to be cost-effective within few years based on rather low improvements of relevant clinical outcomes. Hence, EEE can represent a helpful method to evaluate the economic potential very early in the development process of new innovative solutions based on clinical expert assessments of the potential improvements by the new solution.

### Implications for modern healthcare and future research

These findings are interesting because modern healthcare is becoming increasingly complex with a need for continuous innovative improvements while treating an increasing number of patients. This requires innovative solutions helping to optimise both treatments and everyday workflows. Particularly psychiatry has seen a steady increase in the number of patients, particularly in outpatient settings. Concerning bipolar disorder, the incidence has doubled during the recent two decades (Köhler‐Forsberg *et al*., 2021). Lithium has disadvantages in everyday clinical work due to a narrow therapeutic range and the need for regular 12-h lithium blood levels, with >50% of lithium blood samples not even being within the guideline-recommended window of 10–14 h after intake of the last lithium dose (Jacobsen *et al*., 2025). This has clear clinical implications and can lead to wrong dosing with the risk of increasing side effects or relapse (e.g. when not being aware of the wrong timing), waste of time and resources (e.g. time spend by healthcare professionals on contacting the patient and need for new blood samples), or choice of other treatment (e.g. some patients may not get lithium offered as clinicians expect low compliance with monitoring).

Public payers commonly use economic models in the medicines domain to identify the maximum acceptable price (given ICER [Incremental Cost-Effectiveness Ratio]/WTP thresholds), thereby supporting both coverage decisions and price negotiations (Woods *et al*., 2025). While adoption of these methods has lagged in medtech, the framework is transferable. Here, our model illustrates how the clinical and economic value of a new solution can be quantified and translated into a value-based price range, providing transparent evidence for pricing and reimbursement discussions (Tarricone *et al*., 2017).

Furthermore, EEE can guide future research to optimise studies testing health economic aspects of new innovations. Concerning eLi_12_, an ongoing clinical feasibility study (NCT07306039) was informed by the present EEE approach to measure the potential time savings of lithium implementation (i.e. how many minutes can healthcare professionals save due to eLi_12_) including economic relevant measures that are rarely used in clinical psychiatry (e.g. measure the number of outpatient contacts, bed days, etc.). Hence, by emphasising measures that are of importance for decision-makers in modern healthcare systems, the EEE approach can directly inform clinical studies to include the most relevant measures early in the development process.

### Strengths and limitations

Concerning strengths, the present study applies a transparent decision-analytic framework for early EE in psychiatry, using a payer perspective, NMB, and reporting value-based price and pay-back period. Costs were estimated from national, official sources (medicine prices, DRG tariffs, registries) and uncertainty was explored through scenario and one-way sensitivity analyses, including alternative time horizons and a societal perspective.

Regarding limitations, results from an early, model-based evaluation rely on assumptions and expert input rather than solid evidence from large-scale randomised, controlled trials; the clinical effectiveness of eLi_12_ is not yet established. The 1-year base-case horizon and assumed 100% implementation/uptake may under- or overestimate real-world impact. Generalisability beyond Denmark may be limited by country-specific prices, care pathways, and WTP thresholds. Furthermore, the model does not explicitly capture all downstream consequences of mis-dosing (e.g. adherence, treatment switching, adverse events), which could bias results in either direction. Finally, we did not apply precise register data on the size of the cohort but assumed 28,000 with bipolar disorder and 10,000 treated with lithium. For this overall purpose of early estimations, precise estimates are not needed. The 28,000 is a conservative underestimation, as the actual prevalence of bipolar disorder is estimated to be 1–2%. In general, all numbers and estimates are estimations, which are necessary during such an early phase of development, but have to be interpreted with caution.

### Conclusion

This study illustrates that EEE can represent a helpful approach to evaluate the economic potential of a new solution and guide future research on specific aspects of this solution. The present case eLi_12_ is illustrative as already small improvements in relevant outcomes may result in a good cost-benefit ratio.

## Data Availability

Most of the applied data are published and hence publicly available. The data from the Danish nationwide registers are by Danish law not available for sharing, access has to be granted by the Danish health data authorities.

## Prior presentation

Some of the analyses were presented at the Danish Annual Psychiatric Meeting 2024 in Billund, Denmark, and at the ISPOR 2024 Conference.

## Patient involvement

Patients were not involved in setting the research question, the outcome measures, or the design of the present study.
